# Epigenetically Active Supplements and the Risk of Sports Injuries: Narrative Review from Molecular Mechanisms to Practical Implications

**DOI:** 10.3390/nu18050762

**Published:** 2026-02-26

**Authors:** Agata Leońska-Duniec

**Affiliations:** Faculty of Physical Education, Gdansk University of Physical Education and Sport, 80-336 Gdansk, Poland; agata.leonska-duniec@awf.gda.pl

**Keywords:** epigenetics, musculoskeletal injuries, recovery, physical activity, supplementation, personalized sports nutrition

## Abstract

Background/Objectives Sports-related musculoskeletal injuries remain a major challenge in physically active populations, with substantial interindividual variability in susceptibility and recovery that cannot be fully explained by biomechanics or genetics alone. Epigenetic mechanisms, including DNA methylation, histone modifications, and non-coding RNAs, provide a dynamic interface through which mechanical loading, inflammation, and metabolic signals regulate gene expression during tissue adaptation and repair. This narrative review synthesizes current evidence on “epigenetically active” dietary supplements and their potential relevance to sports injuries, focusing on methyl donors, polyphenols, omega-3 fatty acids, vitamin D, and redox-active nutrients. Methods Targeted searches of PubMed, Scopus, and Web of Science (2000–2026) were performed using epigenetics-, injury-, exercise-, and supplementation-related terms, prioritizing mechanistic and translational evidence. Results Available data indicate that these compounds can influence molecular mechanisms implicated in musculoskeletal recovery. However, human evidence is largely derived from peripheral tissues and indirect molecular markers, with limited clear linkage to clinically significant injury outcomes such as injury incidence, severity, or return-to-play timelines. Accordingly, these nutrients are best viewed as modulators of recovery-related biology rather than as direct therapeutic agents. Conclusions This review highlights a notable translational gap between mechanistic plausibility and clinical evidence and discusses practical implications for sports nutrition from a personalized perspective. Future research priorities include tissue-relevant epigenetic assessments, integration of multi-omics approaches, and longitudinal trials incorporating injury endpoints. Nutritional epigenomics, therefore, represents a promising avenue to support musculoskeletal health while underscoring the need for rigorous clinical validation.

## 1. Introduction

Sports-related musculoskeletal injuries pose a permanent and significant challenge within physically active populations, affecting not only the immediate health and well-being of athletes but also resulting in substantial economic burdens and performance losses [1]. While traditional injury prevention strategies have focused on biomechanical improvement, load management, and rehabilitation, the wide variability observed in individual susceptibility to injury and the rate of recovery suggests that underlying genetic factors play a critical, yet often underestimated, role [2]. Therefore, explaining these inherent determinants can inform the development of more preventive and personalized lifestyle interventions, including improved individual risk profiling, more targeted recovery support, and better alignment of training and rehabilitation strategies with an athlete’s biological response.

Familial and case–control association studies provide consistent evidence that susceptibility to sports-related musculoskeletal injuries has a heritable component, indicating that genetic background contributes to interindividual differences in tissue responses to mechanical loading. Inherited factors may alter key biological processes, such as extracellular matrix (ECM) remodeling, inflammatory responsiveness, and regenerative potential, thereby affecting injury risk and recovery profiles. Importantly, this predisposition appears to be polygenic, reflecting the combined influence of numerous genetic variants rather than a single polymorphism [3,4]. Nevertheless, even comprehensive genomic analyses do not completely explain the substantial inter-individual variability observed in sport-related injury susceptibility and healing outcomes, suggesting that additional regulatory mechanisms influence how genetic potential is translated into athletes’ functional phenotypes. In this context, integrating epigenetic research, focused on DNA methylation, histone modifications, and non-coding RNA regulation, offers a biologically plausible framework to capture load- and environment-responsive modulation of gene expression that cannot be explained by DNA sequence alone [5,6]. For instance, intense physical activity has been linked to alterations in DNA methylation patterns of genes involved in inflammation, muscle repair, and fibrotic remodeling, with responses that may vary substantially between individuals [7]. Together, genomic and epigenetic profiling can therefore provide a more comprehensive and dynamic picture of the biological processes regulating injury risk and regeneration, supporting more individualized strategies in sports medicine [2].

In light of this emerging perspective, the recognition of the epigenome’s plasticity has led to the concept of “epigenetically active” supplementation. These are dietary compounds or supplements that not only provide energy or structural components, but also actively engage with the cellular processes responsible for epigenetic modifications [8,9,10]. Such diet-derived bioactive molecules may act as substrates or cofactors for epigenetic enzymes (e.g., methyl donors for DNA methyltransferases, DNMTs) or directly modulate their activity (e.g., polyphenols inhibiting histone deacetylases, HDACs). The goal of this targeted approach is to promote gene expression patterns that favor tissue integrity, efficient inflammation resolution, and robust regenerative capacity [8].

Despite the widespread use of dietary supplements in sport, existing research has predominantly focused on their ergogenic effects or general support of recovery, with limited attention given to their potential role in modulating molecular mechanisms underlying musculoskeletal injury susceptibility and tissue repair. In particular, although genetic contributions to injury risk have been increasingly characterized, the influence of epigenetically active supplementation on injury development, healing processes, and recurrence remains poorly understood [11,12,13]. The interaction between nutritional compounds, epigenetic regulation, and load-induced tissue adaptation, therefore, represents a relatively novel and underexplored area within sport medicine and exercise science, especially in the context of sports-related musculoskeletal injuries.

Accordingly, this narrative review aims to synthesize current evidence on the role of epigenetically active dietary supplements in reducing the risk of sport-related injuries. The study focuses on epigenetic mechanisms, including DNA methylation, histone modifications, and non-coding RNAs, through which selected nutrients and supplements may influence inflammatory responses, tissue regeneration, and adaptive processes within the musculoskeletal system. Additionally, this review critically evaluates available preclinical and clinical evidence and identifies key research gaps that currently limit the translation of nutriepigenetic findings into sports medicine practice and injury prevention.

## 2. Literature Search and Methodological Approach

This narrative review was informed by targeted literature searches conducted in PubMed, Scopus, and Web of Science. The searches covered publications from January 2000 to 2026, with particular emphasis on recent studies reflecting advances in epigenetic profiling technologies and their translational relevance to musculoskeletal injury and recovery in physically active populations. Search terms were combined using Boolean operators and included keywords related to epigenetics (e.g., DNA methylation, histone modifications, non-coding RNAs), exercise and training (e.g., resistance, aerobic/endurance), musculoskeletal injury and tissue repair (e.g., muscle, tendon, ligament; regeneration, inflammation, extracellular matrix remodeling), and nutritional supplementation (e.g., omega-3 fatty acids, vitamin D, methyl donors, polyphenols). Evidence was prioritized based on mechanistic relevance, methodological rigor, and applicability to athletic contexts, and categorized where possible as direct or indirect with respect to injury-related outcomes. In selected cases, older publications were intentionally included when they provided foundational descriptions of key biological mechanisms discussed in the review. As a narrative review, this work does not follow a formal systematic review framework (e.g., PRISMA) but instead aims to integrate mechanistic and translational evidence to identify clinically meaningful gaps and future research priorities.

## 3. Epigenetic Mechanisms in Musculoskeletal Tissues Relevant to Sports Injuries

The remarkable capacity of musculoskeletal tissues to adapt to mechanical loading and to regenerate following injury is governed by a tightly coordinated network of epigenetic mechanisms that translate external stimuli into context-specific gene expression patterns. These mechanisms allow cells to respond to mechanical stress, inflammation, and metabolic signals, thereby shaping not only the acute injury response but also long-term tissue resilience and susceptibility to reinjury. Importantly, epigenetic regulation represents a biologically plastic interface between environmental exposures and cellular behavior, positioning it as a potentially modifiable layer in the regulation of musculoskeletal health [6,12,14].

DNA methylation is among the most extensively characterized epigenetic modifications in muscle and connective tissues. This process involves the addition of a methyl group to cytosine residues, predominantly within cytosine–phosphate–guanine (CpG) dinucleotides, and has classically been associated with transcriptional repression. However, in the context of musculoskeletal trauma, DNA methylation patterns are highly dynamic and finely regulated. After injury, localized and time-sensitive methylation shifts occur in genes involved in satellite cell activation, inflammatory signaling, and extracellular matrix remodeling, thereby facilitating coordinated tissue repair [7,15,16]. Notably, these changes may persist beyond the resolution of the acute healing phase, resulting in long-term epigenetic reprogramming of muscle stem cells. This epigenetic process has been proposed as a mechanistic basis for skeletal muscle memory and may contribute to altered responsiveness to subsequent mechanical loading, potentially influencing both adaptation capacity and vulnerability to future injuries [14,15,17]. An important translational implication of epigenetic muscle memory is its potential influence on recovery efficiency and reinjury susceptibility. Persistent epigenetic modifications, particularly DNA methylation changes in genes regulating muscle growth, inflammation, and extracellular matrix remodeling, may facilitate more rapid reactivation of regenerative pathways during retraining or rehabilitation. This phenomenon may help explain the accelerated recovery of muscle mass and strength observed in previously trained individuals following injury or disuse. Conversely, incomplete or maladaptive epigenetic remodeling may impair tissue regeneration and increase the risk of reinjury. These findings highlight that prior training history, rehabilitation quality, and environmental factors, including nutritional status, may influence long-term tissue resilience through persistent epigenetic mechanisms. From a rehabilitation perspective, “mechanical loading” should be understood as a qualitatively distinct stimulus. Endurance-type loading preferentially activates metabolic and mitochondrial programs, whereas resistance-type loading more strongly drives myogenic and remodeling pathways. Therefore, the epigenetic “memory” and long-term reprogramming discussed above may be shaped not only by training history, but also by the dominant exercise modality used during return-to-training and reconditioning [14,16,17].

Complementing DNA methylation, histone modifications and chromatin remodeling play a key role in coordinating the temporal sequence of gene expression required for effective tissue repair. Post-translational histone modifications, including acetylation, methylation, and phosphorylation, directly modulate chromatin accessibility and transcriptional capacity [18]. Histone acetylation, in particular, is generally associated with an open chromatin conformation and is crucial for the induction of genes that regulate the resolution of inflammation, angiogenesis, and tissue remodeling during the early and intermediate phases of muscle and tendon healing [19,20]. The precisely regulated balance between histone acetyltransferases (HATs) and HDACs, therefore, represents a crucial control point in determining the efficiency and quality of the regenerative response. Dysregulation of these processes may shift healing and recovery trajectories toward impaired regeneration or excessive fibrosis, outcomes that are highly relevant in the context of repeated sports injuries [21].

Non-coding RNAs, particularly microRNAs (miRNAs), provide an additional, dynamic layer of post-transcriptional regulation in injured musculoskeletal tissues. These small molecules mediate post-transcriptional control of gene expression by targeting specific messenger RNAs for degradation or translational repression, allowing dynamic modulation of important signaling pathways. Following muscle and tendon injury, distinct miRNA expression profiles have been reported and may contribute to the regulation of inflammatory cascades, cell-cycle progression, myogenic differentiation, and extracellular matrix turnover [22,23]. Importantly, these miRNAs act at critical decision points between regenerative healing and fibrotic remodeling, thereby potentially influencing functional recovery outcomes [24,25]. Increasingly, injury- and tissue-specific miRNA signatures are being explored not only as biomarkers of injury severity and healing progression, but also as potential intervention targets to modulate the biological environment toward more favorable repair phenotypes [26,27].

Collectively, these epigenetic mechanisms are consistent with the inherent plasticity of musculoskeletal tissues and may help explain why injury susceptibility and recovery reflect both genetic and environmental influences. They appear to be dynamically shaped by molecular processes that integrate mechanical loading, metabolic status, and inflammatory signals, which may be responsive to targeted modulation. This proposed model provides a biological rationale for exploring interventions, including epigenetically active nutritional strategies, to support tissue integrity and optimize recovery in athletic populations and physically active individuals.

## 4. Nutritional Epigenomics: How Diet and Supplements Influence the Epigenome

The basis of nutritional epigenomics is the assumption that dietary components act not only as sources of energy or structural substrates, but as biologically active environmental signals that can directly influence the genome and its regulatory mechanisms [28]. Through these interactions, nutrients can affect gene expression patterns without altering the underlying DNA sequence, thereby influencing cellular phenotypes involved in adaptation, repair, and stress responses [29,30]. This concept is mainly relevant for tissues characterized by high plasticity and regenerative demand, such as skeletal muscle, connective tissue, and other components of the musculoskeletal system, where epigenetic remodeling has been reported in response to environmental factors such as exercise or diet [31].

Dietary factors exert their epigenetic effects through several interrelated mechanisms. First, nutrients may provide essential substrates required for epigenetic modifications. Methyl-donor compounds, such as folate, vitamin B12, and choline, contribute directly to one-carbon metabolism and the generation of S-adenosylmethionine (SAM), the universal methyl donor for DNA and histone methylation reactions [32]. The availability of these substrates, therefore, directly influences the cell’s capacity to establish and maintain DNA methylation patterns during processes such as cell differentiation and tissue repair [33,34]. Second, certain micronutrients function as cofactors for enzymes that catalyze epigenetic modifications. Minerals and vitamins, including zinc, magnesium, and vitamin D, are obligatory for optimal activity of DNMTs, HATs, HDACs, and other chromatin-modifying enzymes, thereby indirectly modulating chromatin structure and transcriptional activity [35,36]. Third, a range of bioactive dietary compounds can directly modulate epigenetic enzymes or transcriptional regulators. Polyphenols have been shown to inhibit HDACs, while omega-3 fatty acids can influence the activity of transcription factors involved in inflammation and tissue remodeling, linking dietary lipid composition to epigenetic regulation of gene expression [37,38]. These mechanisms suggest that nutrient availability is an important determinant of the cell’s capacity to support the epigenetic reprogramming required for physiological adaptation and effective tissue repair. Rather than acting in isolation, these pathways integrate mechanical and metabolic processes to adjust gene expression in a context-dependent manner [30]. Selected examples of genes and regulatory pathways reported to undergo epigenetic modification in response to nutritional factors relevant to musculoskeletal biology are summarized in Table 1.

Diet and physical activity are among the most powerful lifestyle factors shaping the epigenome, and growing evidence indicates that their effects are often synergistic rather than additive [39]. Exercise itself is a potent epigenetic stimulus, capable of inducing rapid and beneficial changes in DNA methylation and chromatin accessibility in skeletal muscle, including hypomethylation of genes involved in energy metabolism and mitochondrial function [40]. Importantly, the epigenetic signature of exercise is modality-specific, which has direct implications for post-injury recovery and rehabilitation. Aerobic/endurance exercise is consistently associated with epigenetic remodeling of pathways related to oxidative metabolism and mitochondrial biogenesis, including changes in DNA methylation and chromatin accessibility in genes involved in energy metabolism and mitochondrial function. In contrast, resistance exercise more strongly engages epigenetic regulation of myogenesis, hypertrophy-related transcriptional programs, and remodeling pathways supporting force production and structural adaptation. These modality-dependent patterns are clinically relevant because rehabilitation programs often combine endurance-type loading (to restore metabolic capacity and fatigue resistance) with resistance-type loading (to rebuild strength and tissue tolerance), and nutritional strategies may interact differently with each epigenetic program [31,40].

Importantly, in most real-world athletic settings, nutritional interventions should be conceptualized primarily as modulators of the exercise-induced epigenetic landscape rather than independent drivers. In this framework, nutritional interventions may influence the magnitude, timing, or persistence of exercise-induced epigenetic responses rather than initiating entirely independent regulatory programs. Because training elicits large-scale epigenetic remodeling, the independent contribution of supplementation may be subtle and context-dependent, often influencing the magnitude, timing, or persistence of exercise-responsive pathways rather than initiating distinct epigenetic programs. Accordingly, separating nutrition-specific effects from training-induced changes requires carefully controlled designs, including standardized training loads, randomized supplementation protocols, and repeated within-subject sampling; crossover designs, longitudinal profiling, and multi-omic integration may further help distinguish additive, synergistic, or permissive effects [7,14,15,16,17,39,40].

Additionally, experimental evidence suggests that parental physical activity, especially during pregnancy, exerts beneficial effects on maternal and offspring health, partly through epigenetic mechanisms, including DNA methylation changes and modulation of the sperm epigenome [41]. Importantly, these exercise-induced epigenetic modifications are influenced by nutritional status. An optimal, nutrient-dense diet may increase, stabilize, or prolong exercise-driven epigenetic adaptations, thereby supporting a more resilient and adaptive physiological state [31,39,42]. This interaction becomes particularly relevant in the context of musculoskeletal injury and rehabilitation. Injury recovery requires a precisely regulated sequence of gene expression events regulating inflammation resolution, cell proliferation, differentiation, and extracellular matrix remodeling. Controlled mechanical loading during rehabilitation provides a critical epigenetic stimulus, but the successful execution of the repair program also depends on the timely availability of nutritional substrates and cofactors that support epigenetic enzyme function. In this setting, diet and supplementation may influence not only the speed of recovery but also the quality of tissue regeneration and the balance between regenerative healing and fibrotic remodeling [31,39]. This perspective provides a biological justification for examining targeted nutritional strategies to modify epigenetic processes. It also sets the stage for the promising concept of epigenetically active supplementation, which explores the potential of specific dietary compounds to support favorable gene expression patterns during injury prevention and recovery [30].

## 5. Epigenetically Active Supplements with Potential Relevance to Sports Injuries

**Figure 1:**
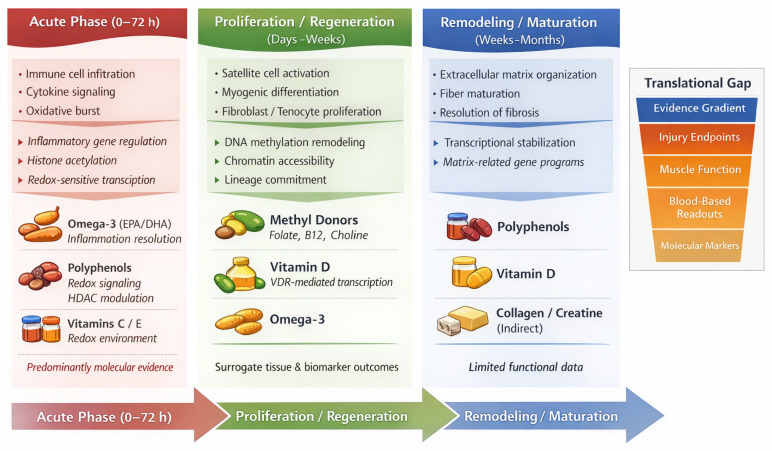
Nutrient–epigenetic targets across phases of musculoskeletal healing. Schematic representation of biological processes and epigenetically relevant pathways during acute inflammation, regeneration, and remodeling, together with nutrient classes proposed to modulate these phases. The evidence gradient illustrates the translational gap between molecular plausibility and clinical injury endpoints.

A growing number of dietary components interact with epigenetic regulatory mechanisms, making them plausible candidates for targeted strategies in sports medicine, injury prevention, and rehabilitation. However, current evidence is considerably stronger for molecular and systemic markers, such as inflammation-related signaling, DNA methylation patterns, and oxidative stress, than for clinical outcomes, including injury incidence and severity, time to return to physical fitness, or reinjury rates. Several nutrient classes appear capable of modulating epigenetic programs relevant to musculoskeletal recovery, particularly those governing inflammation resolution, activation and differentiation of muscle satellite cells and tenocytes, extracellular matrix remodeling, and fibrotic responses. Resolution of inflammation has been associated with epigenetic silencing of pro-inflammatory genes and activation of anti-inflammatory pathways, mechanisms that have been described for n-3 polyunsaturated fatty acids and polyphenols via DNA methylation and histone modifications [38]. Regenerative processes may likewise be influenced by epigenetic control of muscle and connective tissue progenitor cell fate, with methyl-donor nutrients and vitamin D supporting DNA methylation capacity and chromatin-mediated gene regulation relevant to tissue repair [34,36]. In parallel, polyphenols have been linked to epigenetic upregulation of endogenous antioxidant defense systems by modulating histone deacetylase activity and redox-sensitive transcriptional programs, potentially contributing to the control of oxidative stress [43]. These phase-specific interactions and the associated translational gap are summarized schematically in Figure 1.

### 5.1. Methyl Donors

Methyl donors, including folate (vitamin B9), vitamin B12, choline, and betaine, support one-carbon metabolism and the synthesis of SAM, the principal methyl-group donor for DNA and histone methylation. In the context of musculoskeletal injury, this is mechanistically relevant because skeletal muscle regeneration requires coordinated epigenetic transitions that regulate satellite cell activation, lineage commitment, and differentiation [31]. Rather than implying that methyl donors “accelerate healing,” a more defensible interpretation is that they help maintain adequate methylation capacity and support the remodeling of DNA methylation landscapes during regeneration, particularly under conditions of high cellular turnover or differentiation demand [33,34].

Dietary folate is abundant in leafy green vegetables, legumes, and some fruits, whereas vitamin B12 is obtained primarily from animal-derived foods (meat, fish, dairy, eggs) or fortified products. Choline is most concentrated in eggs (especially yolk), meat, fish, and dairy, with smaller contributions from legumes and cruciferous vegetables; betaine is relatively high in beetroot, spinach, whole grains, and certain seafood. From a practical perspective, adequacy is typically discussed relative to established dietary reference intakes (e.g., folate ~400 µg DFE/day and vitamin B12 ~2.4 µg/day for adults; adequate intake for choline ~425–550 mg/day), whereas betaine has no formal recommended intake and is usually considered in relation to habitual diet or study-specific supplementation protocols, given dose-related adverse effects in some settings [44].

In experimental studies, methyl donors are rarely supplemented as isolated epigenetic factors but rather considered part of broader nutritional strategies aimed at preserving one-carbon metabolism under conditions of increased physiological demand [45]. Intervention studies typically employ folate and vitamin B_12_ at doses close to or modestly exceeding recommended intakes, while choline and betaine are more often investigated in the context of performance, body composition, or homocysteine regulation, with supplemental doses commonly ranging from ~500 to 3000 mg/day for betaine and ~500 to 2000 mg/day for choline [46,47,48,49]. Importantly, existing evidence does not support the use of high-dose methyl-donor supplementation as a targeted therapy for musculoskeletal injury per se; instead, its relevance appears to lie in maintaining adequate methylation capacity during periods of high cellular turnover, intensive training, or restricted dietary intake.

### 5.2. Polyphenols

Polyphenols such as resveratrol, curcumin, and quercetin are often described as “epigenetically active” compounds because they can influence chromatin regulation and inflammatory gene expression through multiple, context-dependent mechanisms. The most robust framing emphasizes modulation, rather than uniform inhibition, of epigenetic enzymes and signaling pathways linked to chromatin state, most commonly discussed in relation to histone acetylation/deacetylation dynamics and inflammatory transcriptional programs [38,50]. Through these mechanisms, polyphenols may support endogenous antioxidant defense pathways and promote a more controlled inflammatory response. This is biologically relevant for musculoskeletal injury recovery, as persistent oxidative stress and prolonged inflammation are well-recognized contributors to impaired regeneration and fibrosis-prone tissue remodeling [51,52].

From a dietary perspective, polyphenols are widely distributed in plant-based foods, including fruits and berries (anthocyanins and flavonols), tea and cocoa (flavan-3-ols), coffee and whole grains (phenolic acids), soy products (isoflavones), turmeric (curcuminoids), and grapes or red wine (stilbenes such as resveratrol). Habitual intake in Western populations is typically estimated at approximately 500–1000 mg/day, depending on dietary patterns and consumption of polyphenol-rich beverages such as coffee and tea. Despite this relatively high intake, systemic exposure to parent polyphenols remains limited: only ~5–10% of ingested polyphenols are absorbed in the small intestine, while the majority reaches the colon and is extensively metabolized by the gut microbiota into smaller phenolic derivatives with distinct biological activity. Consequently, circulating concentrations after dietary or supplemental intake generally remain in the low micromolar range [53,54].

In experimental and applied sports nutrition contexts, polyphenols are therefore commonly administered in concentrated or standardized forms at doses exceeding those achievable through habitual diet alone. Commonly investigated ranges in human studies include approximately 80–200 mg/day for curcumin (or higher doses of turmeric extracts standardized in curcuminoids), 100–1000 mg/day for resveratrol-containing preparations, 200–1000 mg/day for quercetin, and 200–500 mg/day for cocoa flavanols, alongside whole-food interventions such as Montmorency cherry juice or berry concentrates. In this context, a shift from uniform antioxidant strategies toward more individualized, food-informed or supplement-informed approaches in sports medicine.

### 5.3. Omega-3 Fatty Acids

Omega-3 polyunsaturated fatty acids (n-3 PUFAs), particularly eicosapentaenoic acid (EPA) and docosahexaenoic acid (DHA), are widely recognized for their anti-inflammatory properties, and accumulating human evidence suggests that they are also associated with changes in DNA methylation within inflammation-related pathways, most commonly assessed in blood-derived immune cells [51,52]. Although extrapolation to injured muscle or tendon tissue must be made cautiously, these findings support a plausible epigenetic link between omega-3 intake and inflammatory regulation. In parallel, mechanistic and review-level evidence indicates that n-3 PUFAs may influence skeletal muscle regeneration indirectly by shaping the inflammatory milieu and satellite cell signaling environment [38]. A careful interpretation is that omega-3 fatty acids may help bias the post-injury environment toward inflammation resolution and regeneration rather than prolonged inflammatory signaling and fibrotic remodeling, while acknowledging that high-quality injury-outcome trials in athletic populations remain limited.

Long-chain n-3 PUFAs are found predominantly in oily fish (e.g., salmon, mackerel, sardines, trout) and to a lesser extent in fortified foods and algal oil supplements; plant sources like flaxseed and chia provide the precursor α-linolenic acid (ALA), which is inefficiently converted to EPA and DHA in humans. Regular consumption of these foods has been linked to increased tissue levels of long-chain n-3 PUFAs, which underlie many of their anti-inflammatory effects. Dietary supplementation with EPA and DHA has been shown to reduce the production of inflammatory compounds in athletes engaged in high-intensity and long-duration exercise, indicating possible relevance for training and recovery settings [55,56].

In sports nutrition research, long-chain n-3 PUFAs are typically administered in supplemental form at doses that exceed what would be achieved through diet alone, with several clinical trials using combined EPA + DHA doses in the range of ~1–3 g/day to investigate effects on systemic inflammation, muscle soreness, and post-exercise recovery biomarkers. For example, ingestion of ~2.4 g/day of combined EPA and DHA for several weeks has been associated with reductions in pro-inflammatory cytokines following eccentric exercise protocols, although consistent effects on performance outcomes remain unclear [57,58]. However, evidence for definitive benefits on functional injury outcomes remains limited [59,60].

### 5.4. Vitamin D

Vitamin D (cholecalciferol) is best conceptualized not simply as a nutrient supporting bone health, but as a nuclear receptor ligand: the active form of vitamin D binds the vitamin D receptor (VDR), which recruits coregulatory complexes and can modify local chromatin structure, thereby influencing transcriptional programs across a wide range of target genes [36]. This mode of action is relevant to skeletal muscle function, immune regulation, and potentially regeneration-related pathways. Observational and interventional studies further link vitamin D status or supplementation to DNA methylation–based aging or biological age acceleration metrics, which are often interpreted as epigenetic correlates of systemic vulnerability rather than direct measures of injury risk [61,62].

Few foods naturally contain substantial vitamin D; the richest sources are oily fish (e.g., salmon, mackerel, sardines), cod liver oil, and fortified products such as dairy or plant milks, while eggs and liver provide modest amounts. Endogenous synthesis in skin via ultraviolet-B exposure is a major contributor to total vitamin D status, and athletes training indoors or at high latitudes are at particular risk of insufficiency or deficiency. Low serum 25-hydroxyvitamin D (25(OH)D) has been associated with reduced muscle strength and increased risk of stress fractures in athletic populations [63,64].

In practice, supplementation with vitamin D is widely used in sports nutrition to achieve and maintain sufficient serum 25(OH)D concentrations, especially in winter months or when baseline levels are low; common regimens in athlete cohorts range from ~1000 to 4000 IU/day to reach target levels (>32–40 ng/mL), though specific protocols vary by individual characteristics and geographic context [63,64]. Clinical studies in athletes suggest that raising serum 25(OH)D may attenuate markers of exercise-induced muscle damage and inflammation and support recovery processes, although effects on performance outcomes are mixed and appear most consistent in individuals with initial deficiency [65,66]. Maintaining adequate vitamin D status is therefore regarded as a practical component of musculoskeletal health and recovery strategies, potentially reducing injury risk and supporting adaptive responses, even if high-dose vitamin D supplementation per se has not been definitively shown to alter injury incidence or return-to-play timelines in well-controlled trials.

### 5.5. Vitamin C, Vitamin E, and Other Potentially Relevant Candidates

Vitamin C and E are most definitely framed as modulators of the redox environment that can secondarily influence epigenetic regulation, rather than as “direct epigenetic supplements”. Vitamin C (ascorbate) is notable because it functions as a cofactor for Fe(II)/2-oxoglutarate–dependent dioxygenases, including ten–eleven translocation (TET) enzymes involved in DNA demethylation and certain jumonji C domain (JmjC) HDACs, providing a plausible route by which vitamin C availability can affect chromatin state and transcriptional programs relevant to differentiation and repair [67,68]. In musculoskeletal contexts, this is conceptually relevant because regeneration and matrix remodeling require coordinated activation/silencing of gene networks, and the demethylation machinery participates in cellular state transitions. Vitamin E (α-tocopherol), in contrast, is better positioned as an antioxidant affecting lipid peroxidation and inflammatory signaling; while not classically epigenetic, oxidative stress is tightly linked to epigenetic maintenance, and redox perturbations can modulate DNA methylation and histone marks indirectly via effects on enzyme activity and cellular metabolite availability [67,68,69,70,71].

From a practical perspective, these vitamins are obtained primarily from fruits and vegetables (vitamin C) and nuts, seeds, vegetable oils, and whole grains (vitamin E), and are commonly consumed at or above recommended intakes in athletic populations. In sports nutrition, antioxidant supplementation has historically been used to attenuate exercise-induced oxidative stress and muscle soreness. However, evidence from experimental studies suggests that chronic high-dose supplementation of vitamins C and E can blunt some skeletal muscle adaptations to training, such as mitochondrial biogenesis and expression of endogenous antioxidant enzymes. For example, combined supplementation of ~500–1000 mg/day vitamin C with ~235–400 IU/day vitamin E has been shown to attenuate training-induced increases in mitochondrial proteins and redox-sensitive signaling pathways in human skeletal muscle. This suggests potential interference with adaptive responses to endurance or resistance training [72,73]. While these effects do not consistently translate to reduced performance outcomes, they align with a broader literature indicating that high-dose antioxidant supplements may dampen beneficial exercise-induced molecular adaptations. Many experts recommend achieving antioxidant adequacy through a diet rich in fruits and vegetables rather than routine high-dose supplementation during periods of intensive training [74,75].

Beyond vitamins C and E, additional candidates sometimes discussed in the context of recovery biology include magnesium and zinc, which function as essential cofactors for a wide range of enzymes, including chromatin-associated proteins such as zinc-dependent HDACs and other regulators of transcriptional activity [35]. In parallel, compounds such as creatine and collagen or gelatin-based strategies are primarily substrate and signaling-oriented rather than epigenetically targeted; however, emerging evidence suggests that they may intersect with gene regulation indirectly through effects on mechanotransduction, cellular energetics, and extracellular matrix remodeling pathways, all of which are tightly coupled to transcriptional and chromatin-level responses during tissue adaptation and repair [76]. In applied sports nutrition, these compounds are typically used to support muscle energetics, connective tissue integrity, or training tolerance rather than to explicitly modify epigenetic regulation.

Collectively, methyl donors, polyphenols, omega-3 fatty acids, vitamin D, and redox-active nutrients (vitamins C and E) can be viewed as elements of a broader nutritional framework that supports epigenetically relevant capacity during musculoskeletal adaptation and repair. Rather than acting as direct therapeutics, these nutrients may bias inflammation resolution, redox homeostasis, and regenerative transcriptional programs [29,30]. Accordingly, “epigenetically active” supplementation is best framed as an adjunct to appropriate mechanical loading and rehabilitation, while acknowledging that causal evidence for athletic injury endpoints remains limited [31,39]. In addition, dose, timing, and duration of supplementation are likely critical determinants of epigenetic responsiveness, yet these parameters remain poorly defined in musculoskeletal tissues. Evidence from human and experimental studies suggests that nutrients such as methyl donors, vitamin D, and omega-3 fatty acids may exert dose-dependent effects on DNA methylation, chromatin accessibility, and inflammatory gene regulation. However, most available data derive from heterogeneous populations, peripheral tissues, or non-injury contexts, limiting direct translation to athletic recovery. Moreover, epigenetic responses may differ between acute and chronic supplementation and may depend on the temporal relationship between supplementation and mechanical loading, highlighting the importance of context-specific dosing strategies [7,31,40].

## 6. Direct vs. Indirect Evidence: What Do We Really Know?

**Figure 2:**
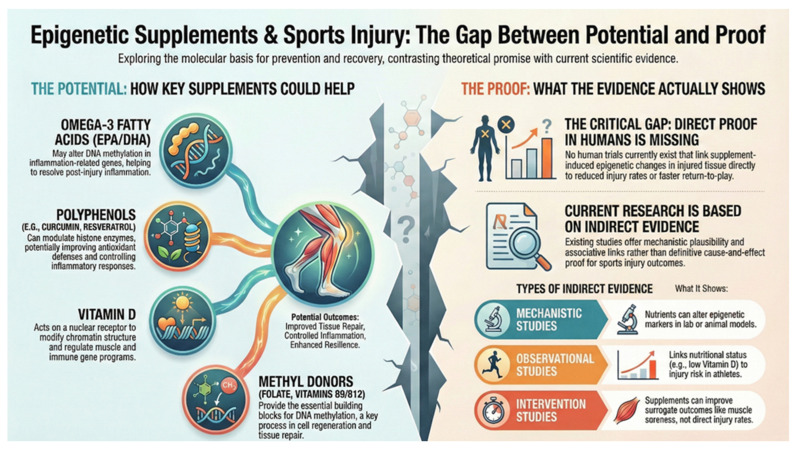
Epigenetically active nutritional supplementation in sports-related musculoskeletal injuries: proposed mechanistic pathways and the translational gap between molecular plausibility and clinical injury endpoints.

Although accumulating studies suggest that nutritionally responsive epigenetic mechanisms influence inflammation, regeneration, and metabolic adaptation, direct human evidence linking supplementation-induced epigenetic changes to musculoskeletal injury outcomes remains scarce. Most available data are derived from peripheral blood or non-injured skeletal muscle and rely on molecular or systemic surrogate markers rather than clinically meaningful endpoints, limiting direct extrapolation to injured tissues and recovery trajectories [31,39]. An overview of representative human studies and their primary molecular targets is provided in Table 2, while Figure 2 summarizes the proposed evidence gradient framework and highlights the translational gap between epigenetic plausibility and injury-related outcomes.

Interpretation is further complicated by substantial heterogeneity across studies, including differences in populations, dosing strategies, intervention duration, tissues examined, and outcome measures. As a result, it remains difficult to determine whether observed epigenetic changes represent causal drivers of tissue repair or downstream correlates of broader physiological adaptation. While these molecular signatures offer important mechanistic insights, they currently provide limited resolution regarding injury incidence, severity, or time to functional recovery [39]. These limitations underscore the need to move beyond molecular associations toward more integrative study designs and clinically relevant endpoints, a perspective that is further developed in the following section by considering translational and methodological priorities for future research.

## 7. Potential Practical Implications for Sports Medicine and Athletic Populations

Although the field of nutritional epigenomics in sports medicine is still evolving, the available mechanistic and human evidence provides a rationale for cautious and informed practical application, particularly within a personalized nutrition framework. Rather than advocating novel or experimental interventions, current insights support refining existing nutritional strategies to ensure that athletes maintain an internal molecular environment conducive to adaptation, recovery, and resilience. This perspective aligns with broader concepts of personalized and precision nutrition, in which dietary strategies are tailored to individual biological needs rather than applied uniformly across populations [30,34].

A first and immediately actionable implication is the prioritization of foundational nutrients that support epigenetic regulation. Sports nutrition practice should ensure adequate intake of key epigenetic cofactors, particularly methyl-donor nutrients involved in one-carbon metabolism (e.g., folate, vitamin B12), vitamin D, and omega-3 fatty acids. These nutrients are essential for maintaining DNA methylation capacity, chromatin regulation, and inflammation control, and deficiencies have been repeatedly reported in athletic and physically active populations, especially for vitamin D and omega-3 fatty acids [59,83]. From a practical standpoint, correcting such deficiencies represents a low-risk intervention that supports fundamental biological processes underlying training adaptation and recovery, irrespective of direct injury outcomes.

Beyond baseline adequacy, targeted supplementation strategies may be considered during periods of elevated physiological stress, such as high training loads, congested competition schedules, or early phases of injury recovery. Bioactive compounds often described as epigenetically active, most notably polyphenols such as curcumin, have been shown to modulate inflammatory signaling and redox-sensitive transcriptional pathways, with indirect links to epigenetic regulation. Strategically timed use of such compounds may help support inflammation resolution and tissue repair processes when endogenous regulatory capacity is challenged, although they should be viewed as adjuncts rather than primary therapeutic agents [84].

Looking ahead, advances in epigenetic monitoring tools raise the possibility of future applications in athlete management. DNA methylation–based biomarkers, including epigenetic clocks and selected methylation panels, have been proposed as indicators of biological age, cumulative stress exposure, and systemic recovery status. While these tools are not yet ready for routine clinical use in sports medicine, early human studies suggest that epigenetic metrics are responsive to lifestyle factors such as diet and physical activity, supporting their potential utility as objective markers of molecular readiness or maladaptation [83,85]. In the longer term, such approaches could complement traditional performance and wellness monitoring to help guide individualized training and nutritional adjustments and reduce the risk of overtraining-related injury.

Taken together, the practical implications of nutritional epigenomics for athletic populations lie not in radical new supplementation paradigms, but in optimizing foundational nutrition, applying targeted strategies during periods of stress, and preparing for future integration of molecular monitoring tools as the evidence base continues to mature.

## 8. Future Directions and Research Agenda

The field of nutritional epigenomics in sports-related musculoskeletal injury is poised for substantial growth; however, its advancement will depend on addressing several critical research gaps. Chief among these is the need to bridge the divide between mechanistic discovery and clinically meaningful application. To date, most evidence remains indirect, underscoring the importance of strategically designed studies that integrate molecular, physiological, and injury-related outcomes.

Disentangling the independent effects of nutritional supplementation from exercise-induced epigenetic adaptations requires carefully controlled study designs. Key methodological approaches include randomized controlled trials with standardized training loads, crossover designs allowing within-subject comparisons, and longitudinal sampling across multiple phases of training and recovery. These approaches help distinguish baseline epigenetic states from dynamic responses to exercise and nutrition. Emerging strategies such as multi-omic integration, including combined analysis of methylation, transcriptomic, and metabolomic profiles, may further improve resolution and help identify additive, synergistic, or permissive roles of nutritional factors within exercise-responsive biological pathways. Such designs are essential for establishing causal relationships and defining clinically meaningful nutritional interventions.

Defining dose–response relationships represents a major priority for future research. Studies should systematically compare physiologic versus supraphysiologic dosing, acute versus chronic supplementation, and supplementation administered before, during, or after injury and rehabilitation. Such designs would help determine whether nutritional interventions influence the initiation, magnitude, or persistence of epigenetic remodeling. Importantly, identifying dose thresholds for beneficial versus negligible effects will be essential for developing clinically relevant, evidence-based supplementation strategies for athletes.

A key research priority is the implementation of longitudinal human randomized controlled trials (RCTs) that simultaneously assess epigenetic markers in relevant target tissues and clinically meaningful injury endpoints, including injury incidence, severity, and time to return to play (RTP). To strengthen translation, future trials should predefine a small set of priority clinical endpoints and the magnitude of change that would be meaningful for athletes and practitioners. Core endpoints should include: (i) injury incidence expressed as injuries per 1000 exposure hours (training and match), (ii) injury severity quantified as time-loss days and proportion of severe injuries, (iii) RTP time using clear medical and performance-based criteria, and (iv) reinjury rates within predefined windows (e.g., 3–6 months post-RTP). Functionally, clinically meaningful recovery should include objective performance metrics such as strength asymmetry indices, range of motion, hop/landing tests where applicable, and sport-specific capacity measures, alongside validated patient-reported outcomes (PROs) using established minimal clinically important difference (MCID) thresholds. Importantly, the meaningful effect size may be modest but highly consequential in practice. For example, a reduction in RTP time by ~5–10%, a reduction in reinjury risk by ~10–20%, or a meaningful improvement in functional symmetry (e.g., achieving ≥90% limb symmetry for relevant strength/performance tests) could materially affect athlete availability, performance continuity, and career longevity. These targets should be interpreted in the context of injury type, competitive level, and baseline nutritional status, and should be paired with tissue-relevant molecular outcomes to directly test whether epigenetic modulation tracks with clinically meaningful recovery trajectories [1,7,40]. Such trials are essential to establish causal relationships between nutritional interventions, tissue-specific epigenetic modifications, and musculoskeletal injury outcomes, and to move beyond inference based on surrogate markers measured in peripheral tissues [85].

To effectively bridge the translational gap, future studies should adopt designs that explicitly separate training-induced epigenetic remodeling from nutrition-related modulation and link molecular changes to clinically meaningful outcomes. Key actionable recommendations include: (i) adequately powered randomized controlled trials with standardized and monitored training loads (including external and internal load metrics) and prespecified rehabilitation protocols; (ii) crossover or within-subject designs where feasible to reduce inter-individual epigenetic variability; (iii) longitudinal sampling across defined phases (baseline/pre-injury where possible, acute post-injury, mid-rehabilitation, return-to-play, and follow-up) to capture dynamic epigenetic trajectories; (iv) careful characterization of baseline nutritional status and diet control to minimize confounding; (v) selection of tissue-relevant biospecimens (e.g., muscle/tendon where ethically feasible, otherwise validated proxy matrices) combined with multi-omic profiling (e.g., methylation, transcriptomics, miRNA) and standardized pre-analytical procedures; and (vi) integration of molecular endpoints with prioritized clinical outcomes (time-loss severity, RTP, reinjury) and functional testing, enabling direct mediation analyses of whether epigenetic changes track with meaningful recovery. Finally, interdisciplinary collaboration between molecular biologists, sports physicians, physiotherapists, and performance staff is essential to ensure ecologically valid interventions and clinically interpretable endpoints [1,7,40].

In parallel, future research should emphasize multi-omics integration, leveraging genomic, epigenomic, transcriptomic, and metabolomic data to construct comprehensive molecular profiles of injury susceptibility and recovery capacity. Conceptual models, such as the gene–environment–epigenome model, provide a conceptual basis for understanding how genetic predisposition interacts with nutritional and mechanical exposures to shape injury risk and resilience, but require empirical validation in athletic populations [86].

Another important direction is the development of non-invasive or minimally invasive approaches to assess musculoskeletal tissue epigenetics. Advances in the analysis of circulating cell-free DNA, extracellular vesicles, and miRNAs in blood offer promising avenues to capture injury- and recovery-related molecular signals without the need for repeated tissue biopsies, thereby improving feasibility in both research and applied sports medicine settings [87,88].

Equally critical is the need to establish dose–response relationships and temporal specificity for epigenetically active nutrients. Future studies should move beyond generic supplementation paradigms to determine the optimal dose, molecular form, and timing of nutrient delivery relative to injury type, training phase, and rehabilitation stage, particularly across the distinct phases of inflammation, proliferation, and remodeling during tissue repair. Such precision is necessary to avoid both underdosing and unintended interference with adaptive training responses [89,90].

In summary, the epigenome serves as a dynamic molecular interface between an athlete’s genetic background and environmental exposures, making it a promising yet incompletely characterized target for nutritional intervention. Although direct clinical evidence linking specific supplementation-induced epigenetic changes to musculoskeletal injury outcomes in humans remains absent, converging mechanistic and human data support the biological plausibility of using epigenetically active nutrients, including methyl donors, omega-3 fatty acids, and polyphenols, to modulate core processes such as inflammation resolution and tissue regeneration [34,38]. Looking ahead, integrating epigenetic insights into personalized nutrition and training strategies could shift sports medicine from a predominantly reactive model to one focused on building molecular resilience and reducing injury risk across the continuum of physical activity. Bridging this translational gap will require coordinated integration of mechanistic insight, tissue-relevant biomarkers, and clinically meaningful injury endpoints in well-controlled athletic cohorts.

## Figures and Tables

**Table 1:** Examples of genes and regulatory pathways epigenetically influenced by selected nutritional supplements relevant to musculoskeletal biology.

Supplement Category	Primary Epigenetic Mechanism	Gene Targets (Examples)	Pathway/Process Targets (Examples)	Evidence Context & Translational Note
Methyl donors	DNA methylation (SAM-dependent)	Interleukin 6 (*IL6*); Tumor necrosis factor (*TNF*); Collagen type I alpha 1 chain (*COL1A1*); Insulin-like growth factor 1 (*IGF1*)	Inflammatory cytokine signaling; ECM remodeling; growth-factor signaling	Evidence largely from peripheral tissues and experimental models; limited injury-specific human musculoskeletal data. Dose/timing for epigenetic effects in athletes remains unclear.
Omega-3 fatty acids	DNA methylation; ncRNA modulation; transcriptional regulation	Peroxisome proliferator-activated receptor gamma coactivator 1-alpha (*PPARGC1A*); Nuclear factor kappa B subunit 1 (*NFKB1*); RELA proto-oncogene, NF-κB subunit (*RELA*); Matrix metalloproteinase 9 (*MMP9*); Matrix metalloproteinase 2 (*MMP2*)	NF-κB-related inflammatory signaling; mitochondrial biogenesis; extracellular matrix remodeling and matrix metalloproteinase regulation	Mixed human/experimental evidence, mostly non-injury contexts; direct confirmation in injured musculoskeletal tissue is limited.
Vitamin D	Chromatin regulation via VDR-mediated transcription	Vitamin D receptor (*VDR*) and downstream VDR-responsive genes; *MMP9*; *MMP2*	Immune modulation; muscle function signaling; tissue repair programs	Heterogeneous populations and contexts; responses may depend on baseline vitamin D status and injury/training phase.
Polyphenols	Histone modification enzyme activity; transcriptional regulation	Sirtuin 1 (*SIRT1*); *MMP9*; *MMP13*	Oxidative stress response; metabolic regulation; inflammatory transcription	Predominantly cell/animal studies; limited direct human musculoskeletal evidence in injury recovery.
Vitamin C	DNA demethylation (TET-dependent)	Ten-eleven translocation methylcytosine dioxygenase 1 (*TET1*); Ten-eleven translocation methylcytosine dioxygenase 2 (*TET2*); Ten-eleven translocation methylcytosine dioxygenase 3 (*TET3*)	DNA demethylation and differentiation programs	Includes disease-specific human cohorts and experimental models; interpret primarily as a mechanistic insight with limited generalizability to healthy athletes.

Most gene-specific epigenetic evidence derives from peripheral tissues, experimental models, or non-injury contexts. Direct confirmation of injury in human musculoskeletal tissues remains limited, and findings should be interpreted primarily as mechanistic insights rather than as clinically established effects.

**Table 2:** Human evidence for epigenetic effects of nutritional interventions potentially relevant to musculoskeletal injury biology.

Intervention	Population	Epigenetic Endpoint	Main Epigenetic Finding	Relevance to Musculoskeletal Injury Biology	References
400 microg folic acid per day for 10 weeks	31 adults with colorectal adenoma (15 supplement, 16 placebo)	Global DNA methylation	↑ global DNA methylation	Supports systemic DNA methylation capacity relevant to inflammatory regulation and cell differentiation during tissue repair	[77]
400 μg folic acid and 500 μg vitamin B12 per day for 2 years	87 elderly participants (44 supplement, 43 placebo)	Gene-specific DNA methylation	Differential methylation at multiple loci, among which genes are implicated in developmental processes	Alters systemic DNA methylation patterns in genes involved in developmental and regulatory pathways, potentially influencing the regenerative environment	[78]
3 g of omega-3 fatty acids per day for 6 weeks	36 overweight and obese adults	Genome-wide DNA methylation	Methylation changes in inflammatory/immune/metabolic genes	Modulates epigenetic regulation of inflammatory and immune-related genes, potentially biasing post-injury responses toward inflammation resolution	[79]
6 g/d Polyphenols (cocoa) for 2 weeks	214 adults with cardiovascular risk (110 supplement, 104 placebo)	Global DNA methylation	↓ global DNA methylation	Influences global DNA methylation linked to redox balance and inflammatory signaling, processes implicated in impaired healing and fibrosis-prone remodeling	[80]
600 IU/d, 2000 IU/d, 4000 IU/d of vitamin D3 for 16 weeks	51 overweight/obese adult males	genome-wide DNA methylation; epigenetic age	changes in DNA methylation and a decrease in epigenetic aging metrics	VDR-mediated chromatin regulation affecting immune and muscle-related gene programs, with potential relevance for injury susceptibility and recovery capacity	[61]
1 g/day vitamin C for 1 year	a family with lymphoma predisposition	genome-wide DNA methylation and gene expression patterns	reinforces the DNA demethylation cascade, reduces the proportion of hypermethylated loci, and diminishes gene expression differences	Enhances DNA demethylation dynamics in humans, supporting epigenetic transitions relevant to tissue remodeling; however, this evidence derives from a disease-specific population and its direct relevance to healthy athletes remains limited	[81]
diet, sleep, exercise and relaxation guidance, and supplemental probiotics and phytonutrients for 8 weeks	43 healthy adult males	Genome-wide DNA methylation, epigenetic clock (DNAmAge)	↓ epigenetic age vs. control	Reduces epigenetic age acceleration, a systemic marker associated with biological resilience and potential vulnerability to injury and impaired recovery	[82]

The relevance statements in Table 2 refer to biologically plausible links between systemic epigenetic modulation and injury-related processes, rather than direct evidence of tissue-specific effects on musculoskeletal injury outcomes. Evidence from non-athletic or disease-specific cohorts (e.g., [81]) is included for mechanistic insight and should not be directly extrapolated to healthy athletes. ↑ means Increase in global DNA methylation; ↓ means Decrease in global DNA methylation.

## Data Availability

No new data were created or analyzed in this study. Data sharing is not applicable to this article.

## Abbreviations

The following abbreviations are used in this manuscript:

ALA	α-linolenic acid
COL1A1	Collagen type I alpha 1 chain
CpG	Cytosine–phosphate–guanine
DNAmAge	DNA methylation age
DNMTs	DNA methyltransferases
DHA	Docosahexaenoic acid
ECM	Extracellular matrix
EPA	Eicosapentaenoic acid
HATs	Histone acetyltransferases
HDACs	Histone deacetylases
IGF1	Insulin-like growth factor 1
IL6	Interleukin 6
JmjC	Jumonji C domain
MMP	Matrix metalloproteinase
MCID	minimal clinically important difference
miRNAs	microRNAs
NFKB1	Nuclear factor kappa B subunit 1
n-3 PUFAs	Omega-3 polyunsaturated fatty acids
PPARGC1A	Peroxisome proliferator-activated receptor gamma coactivator 1-alpha
PROs	Patient-reported outcomes
RCTs	Randomized controlled trials
RELA	RELA proto-oncogene, NF-κB subunit
RTP	Return to play
SAM	S-adenosylmethionine
SIRT1	Sirtuin 1
TET	Ten–eleven translocation
TNF	Tumor necrosis factor
VDR	Vitamin D receptor
25(OH)D	25-hydroxyvitamin D
